# Polycyclic Aromatic Hydrocarbons in *Araucaria heterophylla* Needles in Urban Areas: Evaluation of Sources and Road Characteristics

**DOI:** 10.3390/plants11151948

**Published:** 2022-07-27

**Authors:** Katiuska Alexandrino, Nazly E. Sánchez, Rasa Zalakeviciute, Wilber Acuña, Fausto Viteri

**Affiliations:** 1 Grupo de Biodiversidad Medio Ambiente y Salud (BIOMAS), Universidad de Las Américas, Vía a Nayón, Quito 170124, Ecuador; rasa.zalakeviciute@udla.edu.ec; 2 Departamento de Ingeniería Ambiental y Sanitaria, Universidad del Cauca, Popayan 190007, Colombia; nsanchez@unicauca.edu.co; 3 Departamento de Electrónica, Instrumentación y Control, Universidad del Cauca, Popayan 190007, Colombia; wacuna@unicauca.edu.co; 4 Grupo de Protección Ambiental (GPA), Facultad de Ciencias de la Ingeniería e Industrias, Universidad UTE, Quito 170527, Ecuador; faustor.viteri@ute.edu.ec

**Keywords:** environmental pollution, biomonitor, conifer, PAHs

## Abstract

*Araucaria heterophylla* needles were collected in urban areas of the city of Quito, Ecuador, to analyze the relationship between the concentration of polycyclic aromatic hydrocarbons (PAHs) with different emission sources and road characteristics. The PAHs were analyzed by high-performance liquid chromatography (HPLC) and included naphthalene (Naph), benzo[*a*]anthracene (BaA), chrysene (Chry), and benzo[*a*]pyrene (BaP), which are related to the sources considered in this work. The results indicated that some streets with moderate and low traffic intensity had higher total concentrations of PAHs than streets with high traffic intensity, showing the importance of non-traffic related emission sources and road characteristics on PAH emissions. All the studied PAHs were associated with traffic emissions, although Naph and BaP were more associated with acceleration and braking activities, while BaA and Chry also seemed to come from restaurant emissions. The presence of gas stations was also important in the emission of PAHs. Road capacity seems to have a higher effect on pollutant emission than road gradient and urban forms. The outcomes of this study are expected to facilitate the diagnostics of the concentration of PAHs in urban areas, which contribute to the design of strategies for the mitigation of pollution by PAHs in urban environment.

## 1. Introduction

Polycyclic aromatic hydrocarbons (PAHs) are organic compounds containing two or more fused aromatic rings, which come from incomplete combustion of carbonaceous materials, such as fossil fuels. Many of these compounds have carcinogenic, mutagenic, and teratogenic properties [1,2]; 16 of them have been included in the list of priority pollutants of the Environmental Protection Agency (EPA) [3]. Due to the semi-volatile properties of PAHs, they are partitioned into gas and particle phases once emitted into the atmosphere [4,5]. However, their partitioning depends on different variables such as solubility, temperature, vapor pressure, and the size and surface area of the suspended particles [6].

Road traffic is considered one of the main sources of PAH pollution in urban areas [7,8]. The presence of these pollutants in motor vehicle exhausts is associated with different sources, such as lubricating oil, pyrosynthesis, and unburned fuel [9]. The studies on the association of PAH air pollution with road traffic have been focused on the analysis of particulate matter (PM) and the gas phase. However, the capture of PAHs implies the use of high-cost equipment, which is a limitation factor for low-income countries [10,11].

In general, studies on the contribution of urban sources to the levels of PAHs do not consider non-traffic emission sources, such as cooking emissions [12,13,14] and gas stations [15,16,17]. Moreover, other non-source factors can also influence the levels of PAHs. Several authors have pointed out that high concentrations of pollutants in an area depend on the road geometry and roadside barriers, such as buildings and walls (urban forms). Narrow streets and tall buildings prevent good air ventilation and thus block pollutant dispersion [18,19,20,21,22,23]. Moreover, some studies indicated that road characteristics, such as the road gradient, the number of lanes, traffic lights near the sampling point, roundabouts, intersections, curves, and speed bumps, appear to be relevant for PAH emissions [24,25,26]. Thus, as a first step before defining abatement measures, all these factors should be taken into account when studying the sources and levels of urban PAHs.

Different techniques developed with the aid of scientific observations should be used to define the best road characteristics and benefits for the community [27]. Models are used to establish the effect of different factors, mainly fuel consumption, to predict pollutant emissions [28]. However, due to the high costs of quantifying and measuring air pollutants, the development and validation of urban air pollution models, as important tools for traffic and transit engineers in sustainable road designs [29], are complex and costly tasks.

In this context, a cost-effective way to detect and assess PAH air pollution can be achieved through the use of vegetation. The lipid-rich cuticle of leaves is able to accumulate these pollutants [30]. PAHs in the vapor phase are taken up directly by plants via the stomata, whereas PAHs in the particle phase are accumulated in the lipid-rich cuticle wax layer on the leaf surface [7,31].

In recent years, different plant species [21,32,33,34] have been used to study traffic-related PAH air pollution. In particular, pine has been widely used as a biomonitor due to the high wax content of the needles, which facilitates the accumulation of PAHs over time [35]. Moreover, since pines are evergreens, sampling can be carried out throughout the year [34]. However, further information is needed on the capacity of other conifer species to accumulate PAHs, in order to select the best biomonitor to track PAH contamination in urban areas.

To the best of our knowledge, no study has been conducted using *Araucaria heterophylla* needles for biomonitoring of PAH air pollution, even though it is a widely distributed species in the Southern Hemisphere. It is a dioecious tree with massive erect stems reaching up to 80 m in height, with branches regularly arranged in whorls. This conifer species is widely dispersed in the Andean region and has been used in a previous successful study in the city of Quito, focused on metal air pollution in urban areas with different vehicular traffic intensities [19].

This article describes measurements of the concentration of four PAHs in *Araucaria heterophylla* needles in urban areas with different levels of vehicular traffic intensity in the Andean city of Quito, Ecuador, and relates these to different variables that could affect the level of PAHs, such as the number of restaurants near the sampled trees, urban forms (shallow canyon streets, wide roads, and open roads), road gradient (%), the number of lanes, and speed-modifying devices, which, in this work, included traffic lights, roundabouts, intersections, curves, and speed bumps. The PAHs studied were: naphthalene (Naph), benzo[*a*]anthracene (BaA), chrysene (Chry), and benzo[*a*]pyrene (BaP). Naph was chosen because it is the simplest PAH and is considered one of the first compounds in the chemical formation process of soot, through the well-known HACA mechanism [36]. On the other hand, BaA, Chry, and BaP were chosen because they are commonly analyzed in works studying PAH emissions from cooking [37,38,39]. Moreover, BaA has been found to be predominant near gas stations [16], as well as Chry [15], which has also been found to be predominant in some biomonitoring studies [40,41]. Furthermore, BaP is one of the most potent carcinogens and is the usual marker for carcinogenic levels of PAHs in environmental studies [42,43].

In this way, this study contributes to: (i) expanding the range of vegetation species studied as biomonitors for PAHs, (ii) visualizing the importance of considering other sources of PAH emissions beyond vehicular traffic, and (iii) adding a further step towards obtaining experimental data for modeling studies on the effect of road characteristics on levels of PAHs to contribute to sustainable road design.

## 2. Materials and Methods

### 2.1. Study Area

**Figure 1:**
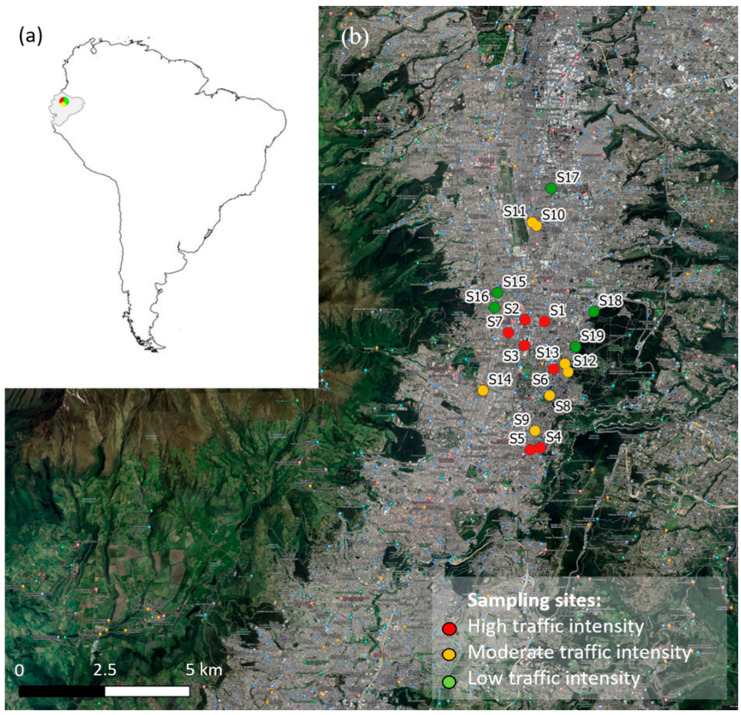
Study area: (**a**) Location of Quito (colored point), Ecuador (gray fill), on the map of South America; (**b**) study sites in Quito.

*Araucaria heterophylla* needles were sampled from 19 sites in Quito (located in the north of Ecuador at 2850 m.a.s.l.). The city has a population size of about 2,239,191 inhabitants [44]. Quito has a spring-like climate for most of the year, with a mean annual temperature of around 13.4 °C, mean annual precipitation of about 1204 mm, and an average annual relative humidity of 76% [45,46]. Figure 1 shows the study area and the sampling points.

The sampling points were those selected by Alexandrino et al. [19], and were classified as high (HTI), moderate (MTI), and low traffic intensity (LTI), according to Google Maps Traffic. Specifically, HTI areas include congested streets with the presence of urban transportation buses and private cars with constant acceleration and braking (red points S1–S7 in Figure 1), MTI areas include streets with slow traffic (yellow points S8–S14 in Figure 1), while LTI areas include streets where vehicular traffic is scarce and there is mainly circulation of private cars (green points S15–S19 in Figure 1).

In the present work, some emission sources were considered for the characterization of the sampling points, such as restaurants near the sampling trees, and some road characteristics, such as urban forms (UF), road gradient (%) (identified from Google Earth), the number of lanes, and speed-modifying devices (traffic lights, roundabouts, intersections, curves, and speed bumps) (Table 1). In the case of a sampling tree placed at an intersection, the lanes of the intersecting streets were added, as was the case for points S8 and S9.

**Figure 2:**
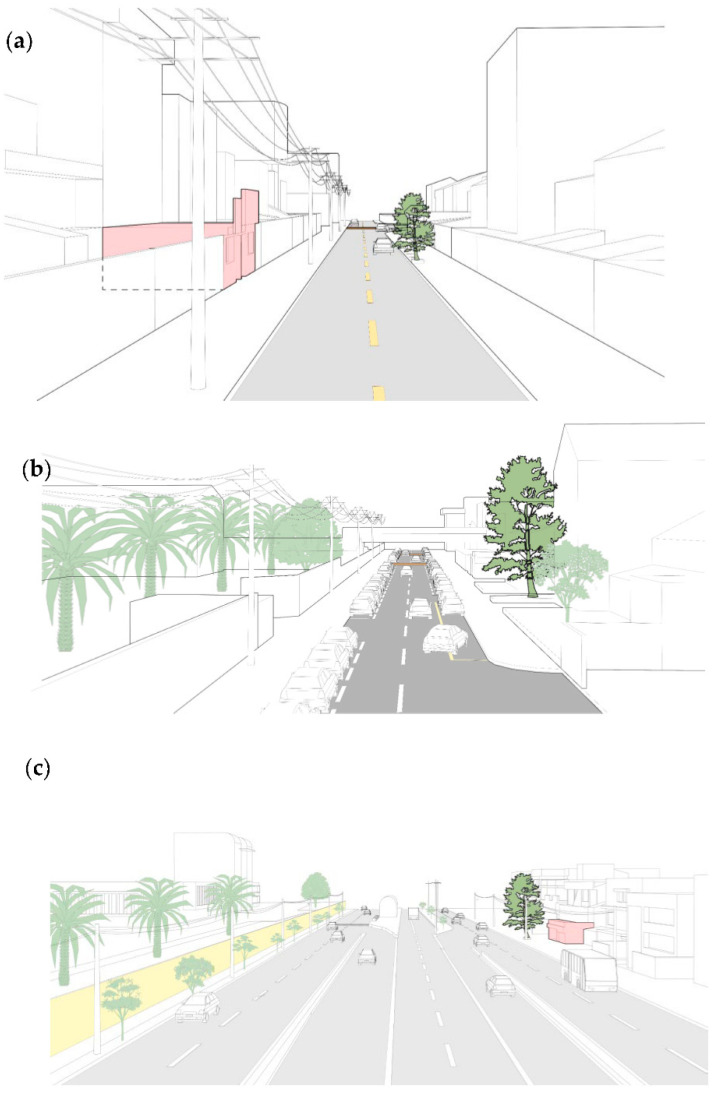
Visual description of: (**a**) a shallow canyon street (Point S19); (**b**) a wide road (Point S7); (**c**) and open road (Point S14), with mobile (cars) and stationary sources (restaurants, in red), and sampled trees bordered in black.

UF refers to the physical characteristics of the streets and their environment, i.e., size, shape, and configuration, as mentioned by Živković [47]. The UF was further classified as shallow canyon streets (SCS), wide roads (WR), and open roads (OR). SCS are characterized by a U-shaped site with narrow platforms, confined between compact rows of shallow houses. Figure 2a shows Point S19 as a representative of a typical street with this shape. WR, represented by Point S7 in Figure 2b, are streets with spacious platforms and open spaces on at least one of their sides and the other with rows of structures, i.e., attached houses and flat buildings. As shown in Figure 2c, with Point S14 as a representative, OR represents streets of vast size, which include a great extent from one side of the road to the other.

### 2.2. Sampling

Sampling was carried out in May 2019, following the procedure described elsewhere [19,48]. No rain was observed during the previous 6 days. At each site, about 8 g of needles with a similar position in the crown was removed from the outer part and in the four directions (north, south, east, and west) of an individual tree, about 2 m above the ground, using plastic gloves; these were pooled in a homogeneous sample. The heights of the trees in the different sampling sites were similar. After sampling, the *Araucaria heterophylla* needles were placed in a clean polyethylene bag, wrapped in aluminum foil, and transported in a cold box to the laboratory. The samples were kept in the dark and frozen until extraction and determination of the percentage of humidity.

The percentage of humidity of the samples was determined by drying, in triplicate, 2 g of the fresh defrosted material at 70 ± 2 °C until a constant weight. In this sense, the results are reported on a dry weight (DW) basis.

### 2.3. Extraction and Chemical Analysis

#### 2.3.1. Solvents and Standard Solution

All solvents (acetone, n-hexane, and acetonitrile) used for sample preparation and analysis were HPLC-grade (Merck, Darmstadt, Germany). Ultrapure water, used in the HPLC gradient, was prepared using a Milli-Q system (Millipore, Burlington, MA, USA). A standard solution of 16 PAHs at 10 μg mL^−1^ in acetonitrile was purchased from Supelco (Quito, Ecuador).

#### 2.3.2. Analytical Procedure

Prior to extraction, fresh samples were defrosted in a desiccator. Next, 2 g of fresh needle samples, cut into 1 cm pieces, were inserted into a beaker and extracted for 10 min with 20 mL of a n-hexane:acetone [1:1 *v*/*v*] mixture in a 420 W ultrasonic bath. This procedure was repeated two more times with fresh solvent. The three extracts were collected into the same round-bottomed flask and concentrated in a Buchi rotary evaporator to approximately 1 mL, and reconstituted with 2 mL of acetonitrile. Next, the solution was filtered with a CHM PVDF syringe filter (0.22 μm) and collected in an amber glass vial to be analyzed.

The instrumental analyses were performed using a HPLC (Agilent 1260 system) equipped with a UV detector (Agilent 1260 DAD G4212B, λ_abs_ = 220 nm, 230 nm, and 254 nm) and a fluorescence detector (λ_exc_ = 254 nm, λ_emi_ = 330 nm). Separation of the PAHs was carried out using a ZORBAX Eclipse PAH column (4.6 × 50 nm, 3.5 µm). The injection volume was set at 20 μL. Elution was carried out at a flow rate of 1.4 mL/min, keeping the column at 25 °C, using the following elution program with acetonitrile (A) and water (B) as the mobile phases: 0–6 min isocratic 40:60 (*v*/*v*) A:B; 6–9.5 min of a linear gradient from 40 to 100% of A and 9.5–12 min of isocratic 40:60 (*v*/*v*) A:B. PAH peaks in the sample chromatograms were identified by comparing the retention time between the standard and sample chromatograms. PAHs were quantified by calculating the concentration of the PAHs with external standard calibration curves, which were obtained with the calibration standard solutions prepared in the concentration range of 5–1000 ng/mL. The correlation coefficients (r^2^) of the calibration curves were greater than 0.993. The analysis of each sample was carried out in triplicate. Chromatograms were processed by ChemStation software (Agilent Technologies, Santa Clara, CA, USA).

In the absence of a certified reference needle material, the accuracy of the method was evaluated with matrix spike recoveries, as has been carried out in different works [49,50,51]. Thereby, 2 g (in duplicate) of a sample of *Araucaria heterophylla* needles was spiked by adding 0.3 mL of a 10 μg mL^−1^ PAH standard mixture until a final concentration of 1.5 μg g^−1^. Spiked and non-spiked samples were prepared and analyzed using the same analytical procedure as the sample. The percentage recovery (%R) was calculated according to the following equation [52]: R = ((concentration of PAHs in the spiked sample − concentration of PAHs in the non-spiked sample)/known added concentration of PAHs in the spiked sample) × 100. Acceptable recoveries (60–130%) were observed for the PAHs studied (Naph, 126%; BaA, 124; Chry, 98%; BaP, 113%). The precision of the method was calculated as the relative standard deviation (RSD) of the concentrations determined in duplicate spiked samples. The %RSD values were below 12%, except for benzo[*a*]pyrene (16.53%).

### 2.4. Geographic Information System

Geostatistical interpolation of the concentrations of PAHs was carried out by using the inverse distance weighted (IDW) interpolation method in QGIS 3.4.4, as has been used in a previous study [19]. This is an interpolation of a point vector (i.e., each PAH) layer with a distance coefficient of 2, which is frequently used to estimate air pollution dispersion in unmeasured regions [53]. During the interpolation, the measurement points were weighted in such a way that the influence of one point relative to other decreased with an increase in the distance from the unknown new point, thus filling in the unmeasured areas.

### 2.5. Statistical Analysis

The interpretation of the data was carried out using principal component analysis (PCA) and multiple correspondence analysis (MCA). PCA is a multivariate statistical tool, which has the main purpose of reducing the dimensional description of the data matrix, facilitating the interpretation of the relationships of the quantitative variables. PCA uses basic geometry to achieve a low-dimensional description of the matrices, considering that the rows of data of Matrix *X* are considered as observations from a p-variate random variable *X*. On this basis, linear combinations are applied to reduce the dimensions of *X* [54]. On the other hand, MCA is used to analyze variables with several levels of nominal data. It could be considered a generalization of PCA when the variables to be analyzed are categorical. MCA is conducted by using component analysis on an indicator matrix.

Quantitative variables, such as the number of restaurants near the sampling trees, the road gradient (%), the number of lanes, speed-modifying devices, and the concentration of individual PAHs were considered as variables in the PCA, while urban forms, categorized PAHs, and lanes, in the nominal form, were considered as variables in the MCA. All statistical analyses were performed using R software through the R commander interface and the Multivariate Exploratory Data Analysis and the Data Mining-FactoMineR package.

## 3. Results and Discussion

### 3.1. Concentration of PAHs in Araucaria heterophylla Needles

**Figure 3:**
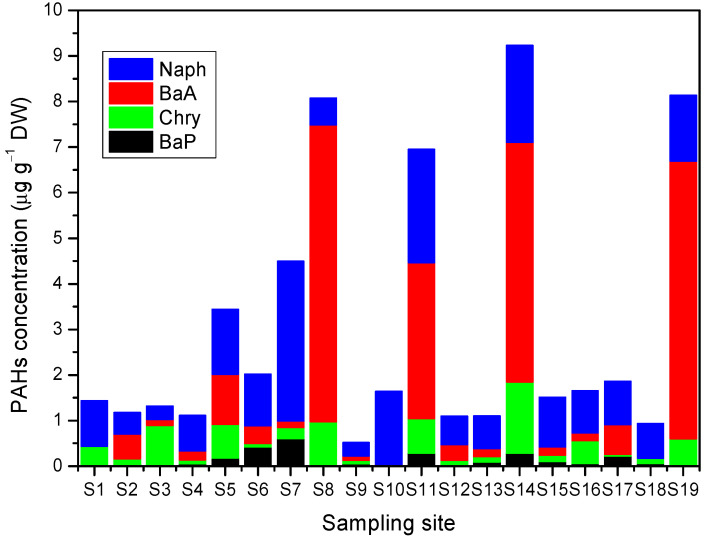
Total and individual concentration of PAHs in needle samples at all sites studied.

The total and individual concentrations of PAHs in the samples at all sites studied are shown in Figure 3. The total concentrations are in the range of 0.52–9.23 μg g^−1^ DW. Figure 3 indicates that, at most of the sites, Naph predominated, followed by BaA, Chry, and BaP, as observed in the works of Fasani et al. [21] and Fellet et al. [42], which were carried out using leaves of the bitter orange tree (*Citrus aurantium*) in streets with different traffic densities, and using the leaves of shrubs growing in public and private gardens exposed to outdoor air pollution, respectively. This result is consistent with the better uptake of the lighter PAHs (2-3-4-rings), which are in the gas phase and can be easily captured by the *Araucaria heterophylla* needles. In contrast, heavy PAHs (five or more rings) are found in particulate matter and can be easily deposited in the soil [55,56].

**Figure 4:**
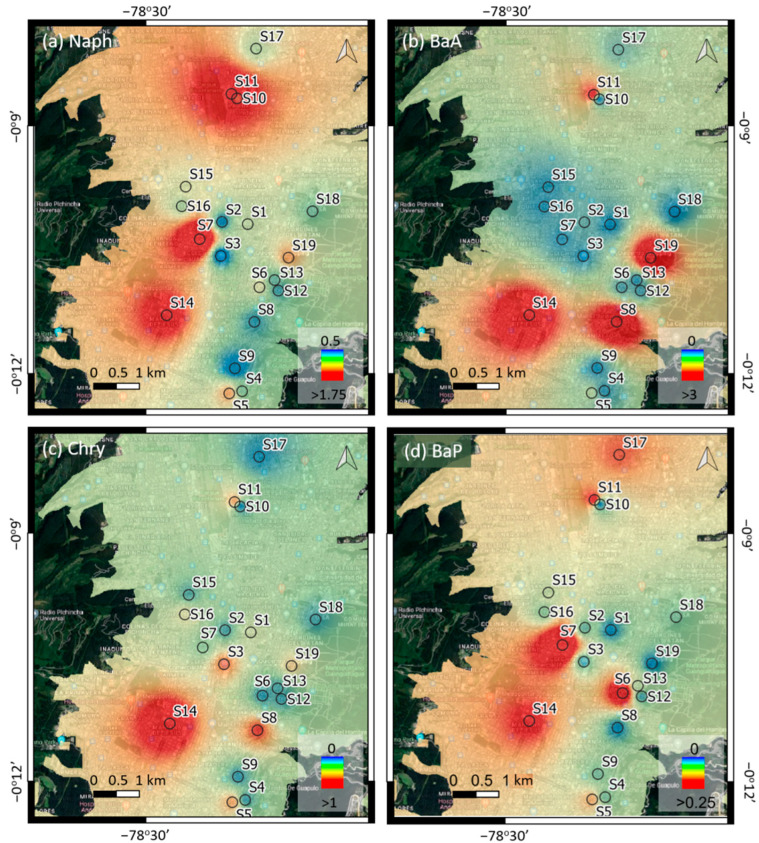
Distribution patterns of the concentration of PAHs studied in the city of Quito.

Figure 4 also shows the distribution patterns of the concentration of each PAH studied. High concentrations of PAHs appear to be scattered throughout the study sites, with the exception of Chry, which shows higher concentrations to the south of the study area. Figure 3 and Figure 4 indicate that the sites with the highest total level of PAHs follow the order S14 > S19 > S8 > S11 > S7 > S5. It is noticeable that even streets with moderate (S14, S8, S11) and low (S19) traffic intensity have a higher total concentration of PAHs than streets with high traffic intensity (S7, S5). A reason for this result could be the existence of non-traffic emission sources and also the road characteristics [14,17,22,24].

S14 was the point with the highest total concentration of PAHs (Figure 3 and Figure 4). It is characterized by being a wide open street with eight lanes (four in each direction) (Figure 2c). There is an underground passage with four lanes, two of which are bus lanes. At this site, the sampling tree is located just in front of the point where the vehicles come from the lower side of the passage to arrive at the upper part, i.e., a point where there are constant acceleration and braking activities. Moreover, the tree is near a traffic light and a roundabout, and is right in front of a Chinese restaurant. Li et al. [13] studied the concentration of PAHs in Chinese, Western, fast food, and Japanese restaurants. They observed that cooking sources contributed to the total PAHs, although they are less important than traffic sources, but have greater carcinogenic potency. Therefore, the high concentration of PAHs at Point S14 may not only be due to vehicular traffic, but also to restaurant emissions, which may contribute to this level of PAHs. In fact, the second point where a higher concentration of PAHs was found, S19 (see Figure 3 and Figure 4), is just in front of a grill restaurant and a fish food restaurant. This is a narrow street with only two lanes (Figure 2a) in a residential area where the traffic intensity is low, but there are compact rows of shallow houses that may hinder the dispersion of pollutants. Along these lines, Rakowska et al. [57] carried out a study on traffic-related gas and particle pollutants in both street canyon and open roadway configurations. They observed that air pollution was lower in a wide and open street with 10 times the traffic volume than in an adjacent street canyon. On the other hand, the high level of PAHs at Point S8 could be due to the narrow shape, the medium traffic intensity, and the relatively high gradient (8.3%), and also because the sampling tree was right on an intersection, being more exposed to emissions from vehicles that slow down and speed up at that point.

S11 is a wide open street with eight lanes, four in each direction, and the sampling point is near an intersection and traffic lights. Moreover, there is a gas station located across the street from the sampling tree that could be contributing to the higher incidence of PAHs. For example, Cobbina et al. [16] carried out a study on the concentration and potential sources of PAHs present in dust particles at gas stations in the Tamale Metropolis, Ghana, in areas with low and high vehicular traffic. They found that petroleum was the largest contributor to PAH emissions in these areas, with a predominance of BaA in both area types. In fact, as seen in Figure 3, at Point S11, the concentration of BaA, was the highest followed by Naph, which has been found to be the dominant PAH in particulate matter emissions from gasoline and diesel vehicles [58,59]. Furthermore, the authors also found that the concentration of PAHs was higher in the areas with low vehicular traffic than in the high-traffic areas, indicating the contribution of other emission sources in urban areas and suggesting the importance of continuing to carry out studies in this regard.

At Point S7, a constant flow of vehicles was observed on a road with closely packed structures on one side of the road that restrict the natural ventilation of pollutants (Figure 2b). Moreover, there are two speed bumps that force drivers to slow down and speed up. On the other hand, Point S5 is close to a roundabout and is located in an area with a high flow of buses that emit a large amount of particulate matter due to the use of low-quality diesel [60]. The high levels of PAHs at Points S7 and S5 are alarming because there are schools and hospitals at these points (Hospital Vozandes at Point S7 and the Baca Ortiz pediatric hospital at Point S5), which could mean that children and sick people are constantly exposed to toxic air pollution. In fact, in our previous work [19], Point S5 was identified as the study site with the highest concentration of traffic-related metals. This shows the importance of taking action to reduce the risk of traffic pollution in these areas, for example, the improvement of fuel and the implementation of new technologies, such as electric buses.

### 3.2. Statistical Data Treatment

**Figure 5:**
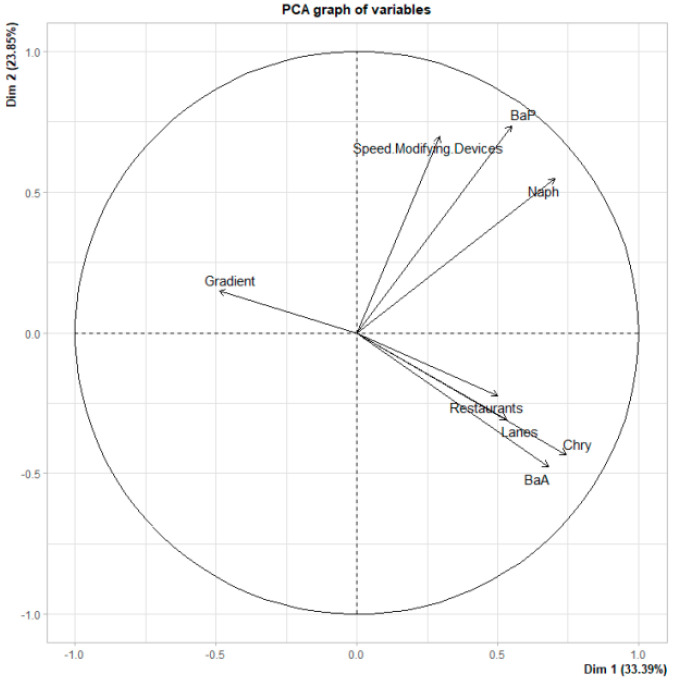
Graph of the variables of the principal component analysis. Naph, naphthalene; BaA, benzo[*a*]anthracene; Chry, chrysene; BaP, benzo[*a*]pyrene.

**Figure 6:**
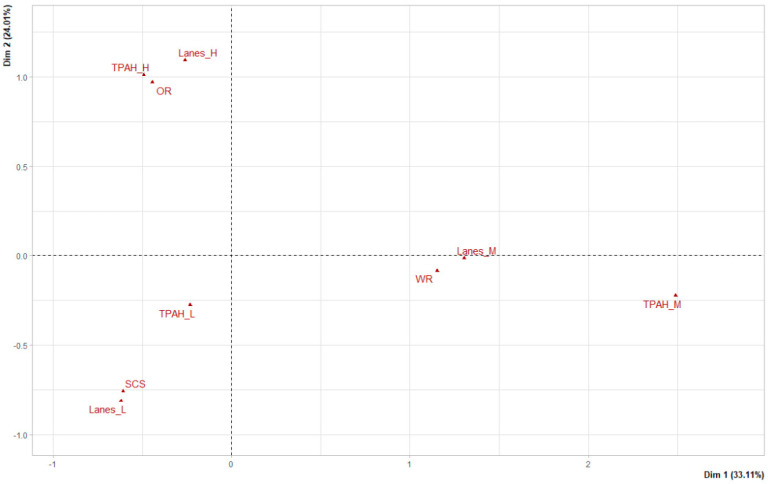
Representation of the variables of the multiple correspondence analysis. TPAH_H, higher total concentrations of PAH; Lanes_H, higher number of lanes; OR, open roads; TPAH_L, lower total concentrations of PAHs; SCS, shallow canyon streets; Lanes_L, lower number of lanes; Lanes_M, medium number of lanes; WR, wide road; TPAH_M, medium total concentrations of PAHs.

The data were statistically interpreted considering the incidence of the sources and the different road characteristics (Table 1) influencing the concentration of PAHs at the sampling points. Figure 5 and Figure 6 include the results of the PCA (association between road gradient (%), the number of lanes and speed-modifying devices, and concentration of individual PAHs) and MCA (association between the urban forms, the categorized lanes, and the categorized total concentration of PAHs), respectively.

The PCA plot (Figure 5) shows that there is a correlation between Naph and BaP, and between BaA and Chry, suggesting the same emission source for each PAH pair. In fact, a close correlation was observed for Naph and BaP with speed-modifying devices, while for BaA and Chry, there is a close correlation with the number of lanes and restaurants. This suggests that all the PAHs studied are associated with traffic emissions, although Naph and BaP are more associated with acceleration and braking activities. Recently, Dhital et al. [24] showed that driving behavior significantly affects fuel consumption and emissions, whereby, for example, a higher mean relative positive acceleration causes an increase in total PAHs. Furthermore, the predominance of certain PAH species could be associated with different formation conditions in combustion or pyrolytic processes, e.g., temperature and residence time [61]. Accordingly, harsh and frequent acceleration/braking with frequent stopping, compared with an almost constant vehicular speed without the presence of speed-modifying devices in roads, could cause different levels of fuel consumption and, consequently, different PAH emission profiles, as suggested by the present work. However, as mentioned by Dhital et al. [24], further research is required to better understand the effects of engines’ operating parameters in accordance with driving behaviors on the profiles of PAH emissions and their emission factors.

BaA and Chry also seem to come from restaurant emissions (Figure 5). In the work of Schauer et al. [62] on the measurement of C1–C27 organic compounds from cooking with seed oils, Chry was the most abundant particulate PAH among the PAHs studied. Moreover, the work of Cordeiro et al. [37], who aimed to determine the effect of different modes of meat marinade used for charcoal-grilled pork on the formation of PAHs, showed that BaA and Chry were the predominant species. Similar findings can be seen in the works of Kafouris et al. [38] and Wang et al. [39].

It can also be observed in Figure 5 that the road gradient is inverse to PAHs, i.e., road gradient does not seem to have a clear effect on the concentration of PAHs, which is opposite to what has been observed in other works. Dhital et al. [24] showed that high slopes increase vehicles’ fuel consumption compared with flat roads and therefore raise the level of PAH emissions. This direct effect seems to be hidden by the road capacity related to the number of lanes. In fact, the number of lanes also seems to hide the effect of urban forms on the concentration of PAHs, as seen in Figure 6. Although an association between the total concentration of PAHs and urban forms can be observed, a lower total concentration of PAHs would be expected to occur on open roads (OR), due to better ventilation, and a higher total concentration on shallow canyon streets (SCS). However, the MCA indicated that this did not occur in this case and instead, a direct association was found between the total concentration of PAHs and the number of lanes, i.e., a greater number of lanes (Lanes_H) is related to a greater total concentration of PAHs (TPAH_H); in addition, a lower number of lanes (Lanes_L) is related to a lower total concentration of PAHs (TPAH_L). Thus, this analysis shows that road capacity hides the effect of urban forms.

The results obtained allow us to see the complexity in the study of pollutants in urban areas where many factors intervene. For this reason, further studies are required to continue identifying the different factors that affect the concentration of PAHs in urban areas, and the impact on human exposure and health risks, and to propose improvements in the formulation of urban planning policies. Trees play an important role as biomonitors to achieve this goal in an easy and cheap way.

## 4. Conclusions

In this work, the concentration of PAHs in *Araucaria heterophylla* needles collected in urban areas with different vehicular traffic intensity, emission sources, and road characteristics was measured. The profile of PAHs associated with *Araucaria heterophylla* needles at different sampling urban sites was consistent with the emission sources and road characteristics, suggesting that this tree species is a good biomonitor of the target PAHs for this work. The data showed that emission sources and road characteristics influenced the level of PAHs beyond traffic intensity. Specifically, the results showed that there was a close correlation of Naph and BaP with speed-modifying devices, such as traffic lights, roundabouts, intersections, curves, and speed bumps, and a close correlation for BaA and Chry with the number of lanes and restaurants. This indicated that even though all the PAHs studied were associated with traffic emissions, Naph and BaP were more associated with acceleration and braking activities, while BaA and Chry were also associated with restaurant emissions. The presence of a gas station near the sampling point also seemed to be important in influencing the level of PAHs. Urban forms seem to be relevant for PAH emissions, although the number of lanes seems to hide its effect, as well as the effect of road gradient. This indicates that the road capacity, measured as the number of lanes on the road, has a greater influence on the total concentration of PAHs. The present study has provided experimental data that suggest the importance of considering non-traffic emission sources and road characteristics when studying the levels of PAHs in urban areas. This information could be of great interest in the design of prevention measures in cities around the world. Additionally, the present work provides useful data to be used in the development of models that contribute to sustainable road designs. Thus, this work reflects the importance of implementing urban planning strategies for urban air quality management in order to reduce the air pollution in urban areas and, in turn, reduce the population’s exposure.

## Figures and Tables

**Table 1:** Emission sources and road characteristics of the sampling points.

Sampling Site	Vehicular Traffic Intensity ^a^	Restaurants	Urban Forms ^b^	Gradient (%)	Lanes	Speed-Modifying Devices
S1	HTI	0	WR	2.5	6	1
S2	HTI	0	SCS	2.3	6	1
S3	HTI	0	OR	1.8	6	1
S4	HTI	0	OR	10.5	4	2
S5	HTI	0	WR	8.5	4	1
S6	HTI	1	WR	10.4	4	2
S7	HTI	0	WR	3.1	4	2
S8	MTI	0	SCS	8.3	4 *	1
S9	MTI	0	WR	6.1	8 *	0
S10	MTI	0	SCS	3.1	2	1
S11	MTI	0	OR	1.4	8	2
S12	MTI	0	SCS	14.3	2	0
S13	MTI	0	OR	7.7	2	1
S14	MTI	1	OR	6.9	8	2
S15	LTI	0	WR	15.8	2	2
S16	LTI	0	OR	7.4	2	2
S17	LTI	0	SCS	5.3	2	3
S18	LTI	0	SCS	16.7	2	2
S19	LTI	2	SCS	5.1	2	1

^a^ HTI: high traffic intensity; MTI: moderate traffic intensity, LTI: low traffic intensity. Classification according to Alexandrino et al. [19]. ^b^ SCS: shallow canyon streets; WR: wide roads; OR: open roads. * Lanes of the intersecting streets were added.

## Data Availability

The data presented in this study are available on request from the corresponding author.
